# History and future perspectives of adipose tissue macrophage biology

**DOI:** 10.3389/fphar.2024.1373182

**Published:** 2024-03-18

**Authors:** Tomonobu Kado, Ayumi Nishimura, Kazuyuki Tobe

**Affiliations:** First Department of Internal Medicine, Graduate School of Medicine and Pharmaceutical Science, University of Toyama, Toyama, Japan

**Keywords:** macrophage, adipose tissue macrophage, adipose tissue, obesity, diabetes

## Abstract

Macrophages contribute to adipose tissue homeostasis; however, they are also thought to be responsible for insulin resistance in obesity. Macrophages, which were oversimplified in past methodologies, have become rather difficult to understand comprehensively as recent developments in research methodology have revealed their diversity. This review highlights recent studies on adipose tissue macrophages, identifies controversial issues that need to be resolved and proposes a scenario for further development of adipose tissue macrophage biology.

## 1 Introduction

In 1986, Mosmann and Coffman had found that T cells could be classified into two types: Th1 and Th2 cells (Mosmann et al., 1986). Th1 cells produce cytokines, such as IFNγ, and activate macrophages, which in turn produce NO (Mills et al., 2000). Th2 cells produce IL-4, IL-5, and IL-13 and inhibit macrophage activation (Romagnani, 1999; Mills et al., 2000). Th1 cells eliminate intracellular parasitic bacteria and viruses, whereas Th2 cells defend against helminth parasites (Romagnani, 1999). The M1/M2 paradigm, which refers to the Th1-type response of macrophages as M1 and Th2-type response as M2, was proposed by Mills et al., in 2000 (Mills et al., 2000). Since then, macrophages have been shown to polarize into M1/M2 macrophages. However, recently, the widespread use of single-cell and single-nucleus RNA-seq has revealed the existence of diverse macrophages that cannot be distinguished simply by M1/M2 (Hill et al., 2018; Jaitin et al., 2019; Silva et al., 2019; Félix et al., 2021; Magalhaes et al., 2021; Sárvári et al., 2021; Emont et al., 2022). In addition, macrophages increase rapidly with obesity and are thought to be responsible for chronic inflammation associated with obesity (Weisberg et al., 2003; van Beek et al., 2015). A common theory is that the number of inflammatory macrophages increases in obesity, leading to insulin resistance (Weisberg et al., 2006; van Beek et al., 2015). Moreover, adipose tissue macrophages in obesity are diverse beyond the inflammatory perspective (Zeyda et al., 2007; Wentworth et al., 2010; Hill et al., 2018; Jaitin et al., 2019; Silva et al., 2019; Félix et al., 2021; Magalhaes et al., 2021; Sárvári et al., 2021; Emont et al., 2022; Muir et al., 2022). Further understanding of macrophages may provide new therapeutic targets for obesity and diabetes. This review presents an overview of adipose tissue macrophage research, including its history and the latest findings.

## 2 Pro-inflammatory or anti-inflammatory

**FIGURE 1:**
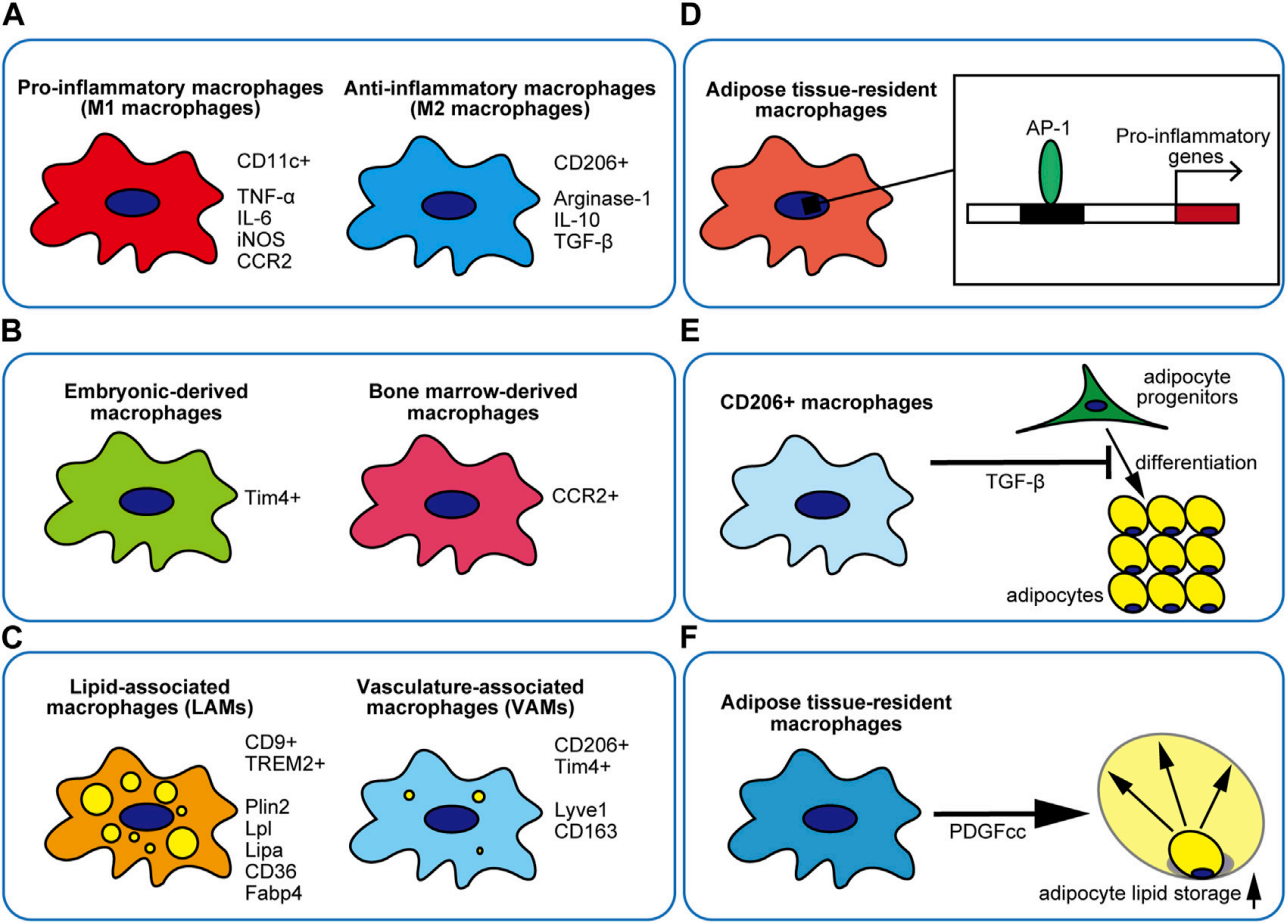
Classification of adipose tissue macrophages, including newly identified subtypes.

In 2007, macrophages were classified as M1/M2 in obese adipose tissue (Lumeng et al., 2007). M2 macrophages are characterized by high surface expression of CD206 and high gene expression such as Arginase-1 (Fujisaka et al., 2016). M2 macrophages are predominant in the adipose tissue of lean mice, whereas M1 macrophages that highly express surface markers, such as CD11c, are increased in obese adipose tissue (Lumeng et al., 2007; Fujisaka et al., 2016). Anti-inflammation-related gene expression such as IL-10 and TGF-β is higher in M2 macrophages, whereas inflammation-related gene expression such as TNF-α and iNOS is higher in M1 macrophages and the expression levels of genes that are highly expressed in M2 macrophages are reduced (Lumeng et al., 2007; Fujisaka et al., 2009; Fujisaka et al., 2016). The removal of CD11c^+^ macrophages reduces the expression levels of genes and proteins for inflammatory markers in the adipose tissue and improves insulin sensitivity and glucose tolerance (Patsouris et al., 2008). These results have led to the use of CD11c as an adipose tissue M1 macrophage marker. Obese adipose tissue macrophages have been explained by the polarization of anti-inflammatory CD206^+^ M2 macrophages or pro-inflammatory CD11c^+^ M1 macrophages (Lumeng et al., 2008; Fujisaka et al., 2009) (Figure 1A). Increased numbers of CD11c^+^ M1 macrophages have been reported to be induced in adipose tissue by monocytes recruited *via* the monocyte chemotactic protein 1/C-C chemokine receptor type 2 (MCP1/CCR2) pathway in an obese state (Lumeng et al., 2007). In obese CCR2 KO mice, macrophage accumulation was found to be reduced, adipose tissue inflammation was decreased, and insulin sensitivity was improved (Weisberg et al., 2006). In obese MCP1 Tg mice, macrophage accumulation was found to be increased and glucose tolerance and insulin resistance were exacerbated (Kamei et al., 2006; Kanda et al., 2006). Moreover, macrophages have been shown to accumulate around necrotic adipocytes in obese adipose tissue and are thought to remove adipocyte debris (Cinti et al., 2005). Histological structures composed of necrotic adipocytes surrounded by macrophages are known as crown-like structures (CLSs) (Cinti et al., 2005). CCR2^+^ macrophages with high expression of M1 markers and low expression of M2 markers have also been reported to accumulate around necrotic adipocytes (Lumeng et al., 2008). However, subsequent studies showed that the M1/M2 paradigm alone cannot explain the diversity of adipose tissue macrophages. Several studies have reported that the classical M1 macrophage inducer lipopolysaccharide (LPS) suppresses CD11c expression (Becker et al., 2012), CD206^+^ macrophages express IL-6 and IL-8, which are classified as pro-inflammatory cytokines (Muir et al., 2022), and CD206^+^ macrophages do not express higher levels of M2-like genes than CD11c^+^ macrophages, which cannot be explained by the M1/M2 paradigm alone (Catrysse et al., 2021). The presence of M1/M2 mixed-type macrophages has also been reported (Zeyda et al., 2007; Wentworth et al., 2010). Kratz et al. proposed a new type of macrophages called metabolically activated macrophages (MMe macrophages), which are distinct from both M1 and M2 macrophages (Kratz et al., 2014). MMe macrophages were stimulated with Glucose, Insulin, and Palmitate *in vitro* (Kratz et al., 2014). MMe macrophages differ from classical M1 macrophages, even though they have elevated levels of inflammation-related genes (Kratz et al., 2014). MMe macrophages expresses PPARγ and P62 along with IL-1β and TNF-α and can well mimic adipose tissue macrophages *in vivo* in the obese state (Kratz et al., 2014). Subsequently, MMe macrophages *in vivo* were shown to highly express pro-inflammatory cytokines and induce insulin resistance (Coats et al., 2017). NADPH oxidase 2 (NOX2) was also a key driver of pro-inflammatory cytokines in MMe macrophages (Coats et al., 2017). In detail, macrophage-specific PPARγ-deficient mice showed an increase in M1 macrophages and a decrease in M2 macrophages in obese adipose tissue, leading to insulin resistance (Odegaard et al., 2007). Furthermore, blocking PPARγ with a PPARγ antagonist increased IL-1β expression in MMe macrophages (Kratz et al., 2014). Insulin resistance was induced in p62−/− mice and IL-1β and TNF-α were highly expressed in macrophages in p62−/− mice (Kratz et al., 2014). These findings suggest that PPARγ and p62 inhibit pro-inflammatory cytokine production by macrophages. Thus, the M1/M2 paradigm was appropriate for roughly characterizing obese adipose tissue macrophages; however, as research in this area progressed, it became clear that it was too simplistic.

## 3 Origin of adipose tissue macrophages

Macrophages present in tissues under steady-state conditions are called tissue-resident macrophages. The origin of tissue-resident macrophages is debatable (Epelman et al., 2014; Hoeffel and Ginhoux, 2018; Wu and Hirschi, 2021; Mass et al., 2023). Primitive hematopoietic progenitors that appear in the yolk sac (YS) on embryonic day 7 (E7) are primitive macrophages that are the origin of microglia (Hoeffel and Ginhoux, 2018). Subsequently, erythromyeloid progenitors (EMPs) appear in the hemogenic endothelium (HE) of E8.25 to YS and generate fetal monocytes (Hoeffel and Ginhoux, 2018). Fetal monocytes migrate to all tissues except the brain, where they become tissue-resident macrophages (Hoeffel and Ginhoux, 2018). Hematopoietic Stem cells (HSCs) also appear at E10.5 HE in the aorta, umbilical artery (UA), and vitelline artery (VA) regions (Hoeffel and Ginhoux, 2018). HSCs colonize the fetal liver, a major hematopoietic organ, to produce fetal monocytes (Wu and Hirschi, 2021). Monocytes derived from the fetal liver are also known as tissue-resident macrophages (Hoeffel and Ginhoux, 2018). In particular, the majority of Kupffer cells in the liver and Langerhans cells in the skin are well known to be of yolk sac or fetal liver origin (Epelman et al., 2014). Tim4, a phosphatidylserine receptor, is present in many tissue-resident macrophages, including adipose tissue, and has been reported to be a marker of fetal-derived macrophages (Chen and Ruedl, 2020). Tim4^+^ macrophages are long-lived and are less likely to be replaced by monocyte-derived macrophages (Chen and Ruedl, 2020) (Figure 1B). Magalhaes et al. showed that F4/80^hi^ Tim4^+^ adipose tissue macrophages, when compared to F4/80^hi^ Tim4^-^ adipose tissue macrophages and F4/80^low^ adipose tissue macrophages, are increased during high-fat diet feeding, not by an increase in monocyte-derived macrophages, but by the proliferation of tissue-resident macrophages (Magalhaes et al., 2021). Félix et al. reported that CD206^+^ Tim4^+^ CD163^+^ adipose tissue macrophages are of embryonic origin (Félix et al., 2021).

After birth, hematopoiesis switches from the fetal liver to the thymus and bone marrow, and the bone marrow becomes the primary source of all blood cell lines, including circulating monocytes (Kloc et al., 2023). Macrophages, which are increased in adipose tissue during obesity, have also been reported to be induced in adipose tissue by bone marrow-derived monocytes in an MCP1/CCR2 pathway-dependent manner (Kamei et al., 2006; Kanda et al., 2006; Weisberg et al., 2006; Lumeng et al., 2008) (Figure 1B). These are CD11c^+^ inflammatory macrophages and reducing the accumulation of these macrophages in CCR2 KO mice improved insulin sensitivity (Weisberg et al., 2006; Lumeng et al., 2008). Lipid-associated macrophages (LAMs), which have become a hot topic in recent years, are mostly of monocytic origin (Hill et al., 2018; Jaitin et al., 2019; Stansbury et al., 2023). Whether the origin of macrophages or the tissue niche is important for determining the characteristics and function of adipose tissue macrophages is not yet well defined and warrants further study.

## 4 Lipid-associated macrophages (LAMs)

Hill et al. found that CD9^+^ macrophages are increased in obese mouse adipose tissue, and that these macrophages are lipid-rich, pro-inflammatory macrophages present in CLSs (Hill et al., 2018). CD9^+^ macrophages are derived from hematopoietic cells and exhibit elevated expression of proinflammatory, lysosomal, and lipid metabolism-related markers (Hill et al., 2018). Jaitin et al. found that circulating monocyte-derived lipid-rich TREM2^+^ macrophages increased in the adipose tissue in obesity. They reported that Trem2 deficiency inhibited macrophage mobilization in obese adipose tissue, causing adipocyte hypertrophy, hypercholesterolemia, inflammation, and glucose intolerance (Jaitin et al., 2019). These TREM2^+^ and CD9^+^ macrophages are termed lipid-associated macrophages and are associated with lipid metabolism (Jaitin et al., 2019) (Figure 1C). Silva et al. reported that the resident macrophages in healthy adipose tissue are in close contact with blood vessels and have a high endocytic capacity (Silva et al., 2019). These macrophages are called vasculature-associated adipose tissue macrophages (VAMs) (Silva et al., 2019) (Figure 1C). In contrast, adipose tissue macrophages during obesity have an increased monocyte-derived CD11c^+^ CD64^+^ macrophage population, with predominant anti-inflammatory/detoxification gene expression but reduced endocytic capacity (Silva et al., 2019). These CD11c^+^ CD64^+^ macrophages expressed high levels of anti-inflammatory genes, such as IL-10 and TGF-β, which differs from the classical view that adipose tissue macrophages, which are increased during obesity, are pro-inflammatory (Silva et al., 2019). Sarvari et al. used single-nucleus RNA-seq (snRNA-seq) to analyze adipose tissue macrophages (Sárvári et al., 2021). Their analysis also identified macrophage subpopulations in adipose tissue that were similar to VAMs and LAMs (Sárvári et al., 2021). Few LAMs were observed in lean state; however, obesity resulted in the largest population of LAMs (Sárvári et al., 2021). In VAMs, VAM-specific gene expression (Csfr1, Cd163, etc.) decreases with obesity, whereas the expression of genes involved in lipid storage increases. This suggests that the gene profile of VAMs is similar to that of LAMs during obesity (Sárvári et al., 2021). In addition, a study using spatial transcriptome analysis showed that LAMs are abundant in CLSs (Stansbury et al., 2023).

Conflicting theories have reported that LAMs are inflammatory macrophages that exacerbate metabolism and non-inflammatory macrophages that are beneficial to metabolism. While some claim that Trem2 deficiency causes adipocyte hypertrophy and insulin resistance (Jaitin et al., 2019), Nathan et al. concluded that Trem2 deficiency causes adipocyte hypertrophy but not insulin resistance and that the differences in glycemic control reported in Trem2^−/−^ mice can be explained by differences in body weight or fat mass (Winn et al., 2022). They concluded that Trem2 deficiency was unlikely to exacerbate glucose homeostasis in mice. A discussion is also needed on whether weight-aligned comparisons should be made or whether differences in weight should also be considered an important phenotype. Conclusions on whether LAMs can be considered as a population that prevents insulin resistance are too early to reach. Trem2 is also expressed in liver macrophages, and the macrophage-specific loss of Trem2 has been reported to exacerbate NASH (Wang et al., 2023), making it difficult to explain the function of LAMs alone in a model of systemic Trem2 loss. Whether LAMs can be considered a population that prevents insulin resistance should be determined after analysis in genetically engineered mice, in which LAMs other than TREM2^+^ LAMs are specifically removed.

In humans, CD9^+^ macrophages are abundant in CLSs and contain high levels of intracellular lipids (Hill et al., 2018). The number of CD9^+^ macrophages is positively correlated with BMI (Hill et al., 2018). Furthermore, the proportion of TREM2^+^ macrophages positively correlates with BMI (Jaitin et al., 2019). Therefore, these findings confirm that CD9^+^ and TREM2^+^ macrophages are increased in obese humans and mice. However, their functions in humans remain unclear.

## 5 Epigenetic reprogramming of adipose tissue macrophages

Hata et al. found that adipose tissue macrophages are retained in a state that facilitates the production of proinflammatory cytokines even when mice become obese and lose weight. Stearic acid increases the expression of transcription factor AP-1 *via* TLR4 (Hata et al., 2023). Binding of AP-1 to DNA mobilizes histone acetyltransferase P300, which acetylates histone proteins, promotes chromatin opening, and upregulates the expression of inflammation-related genes, resulting in epigenetic reprogramming (Hata et al., 2023) (Figure 1D). Hill et al. reported that inflammatory transcription factor motifs including AP-1 and NF-κB are enriched in CD9^+^ adipose tissue macrophages after a high-fat diet (Hill et al., 2018). This suggests that CD9^+^ adipose tissue macrophages contain epigenetically reprogrammed populations in which inflammation-related gene expression is more likely to be elevated. Nonetheless, certain questions remain, such as how long macrophages undergoing epigenetic reprogramming will survive and persist, and whether any factors other than TLR4 stimulation can cause epigenetic reprogramming of macrophages.

## 6 Interaction between adipose tissue macrophages and adipocytes

Nawaz et al. reported that the deletion of CD206^+^ macrophages resulted in smaller adipocytes and improved insulin sensitivity (Nawaz et al., 2017). In addition, a decrease in TGF-β signalling has been shown to lead to an increase in small adipocytes with an increase in adipocyte progenitors (Nawaz et al., 2017) (Figure 1E). Cox et al. reported that the platelet-derived growth factor (PDGF)/vascular endothelial growth factor (VEGF)-related factor 3 (Pvf3) ortholog produced by adipose tissue-resident macrophages increases lipid storage in adipocytes in response to the diet (Cox et al., 2021) (Figure 1F). In contrast, blocking PDGFcc reduces lipid storage in adipocytes, and the unstored lipids are redirected to increase thermogenesis in brown adipose tissue (Cox et al., 2021).

## 7 Clinical applications

Macrophage polarity and macrophage subtypes are associated with insulin resistance, adipocyte differentiation, and lipid storage in adipocytes. In addition, macrophages account for approximately 50% of the immune cells in the adipose tissue during obesity (Weisberg et al., 2003), supporting their validity as a therapeutic target. Developmental potential is expected for drugs that improve insulin resistance and are more effective than existing drugs. Moreover, targeting highly specific macrophages that do not exist in other tissues may reduce the side effects.

Although many basic studies have been conducted on the potential of macrophage-targeted anti-obesity and anti-diabetic drugs, these drugs have not yet reached the stage of becoming products for widespread clinical use. Regulating macrophage polarity changes, inflammation suppression and adipocyte differentiation is necessary if the main target is to ameliorate insulin resistance. PI3K/Akt signaling (Vergadi et al., 2017), KLF4 (Liao et al., 2011), PPARγ (Odegaard et al., 2007), and STAT6 (Zheng et al., 2015) for macrophage polarity changes; TLR4 (Saberi et al., 2009), IKKβ (Arkan et al., 2005), and MyD88 (Yu et al., 2014) for inflammation; CD206 (Nawaz et al., 2017) for adipocyte differentiation are potential targets. The target should be a product that has the potential to be superior to many existing drugs, not simply in losing weight or lowering blood glucose, but also in improving the complications of diabetes and obesity.

Remarkable progress has been made in the genetic manipulation of macrophages. Chimeric antigen receptor (CAR) T-cell therapies are gradually being used in cancer therapy, and the antitumor effects of CAR macrophages have been recently confirmed (Wang et al., 2022). Lei et al. constructed a tandem CD3ζ-TLR4 intracellular toll/IL-1R (TIR) dual signaling CAR and successfully polarized macrophages to M1 in an NF-κB pathway-dependent manner (Lei et al., 2023). CRISPR-dependent transcriptional activation or inhibition systems, such as CRISPRa (CRISPR activation) and CRISPRi (CRISPR interference), have also been developed (Bendixen et al., 2023). These genetic modulation techniques can be applied for gene therapy targeting adipose tissue macrophages.

## 8 Conclusion

Developments in single-cell-level analysis methods, such as snRNA-seq and spatial transcriptomics, have revealed the diversity of macrophages. However, although the diversity of macrophages at the genetic level is becoming clearer, the functional aspects of each are still not fully understood. In the future, identifying specific markers that represent each of the multiple categories of macrophages and using gene editing will be essential to gain a better functional understanding of macrophages. In particular, further research is expected to progress on cutting-edge topics, such as macrophage interactions with other cells, macrophage aging, epigenetic regulation of macrophages, macrophage metabolism, and the development of methods to artificially modulate macrophage function.
